# Orbital Myeloid Sarcoma: An Initial Presentation of Acute Myeloid Leukemia With Maturation

**DOI:** 10.7759/cureus.77580

**Published:** 2025-01-17

**Authors:** Jordy Batista, Paula L Matías, Virgilio Valerio, Luisa Collado, Félix Contreras Mejuto

**Affiliations:** 1 Pathology, Laboratorio de Patología Contreras Robledo, Santiago de los Caballeros, DOM; 2 Ophthalmology, Hospital Regional Universitario Jose María Cabral y Báez, Santiago de los Caballeros, DOM; 3 Pediatric Oncology, Hospital Infantil Regional Universitario Dr. Arturo Grullón, Santiago de los Caballeros, DOM; 4 Pathology, Pontificia Universidad Catolica Madre y Maestra/Clinica Universitaria Union Medica, Santiago de los Caballeros, DOM

**Keywords:** acute myeloid leukemia (aml), extra-medullary acute myeloid leukemia, myeloid sarcoma, ocular chloroma, orbital myeloid sarcoma

## Abstract

Acute myeloid leukemia (AML) is predominantly an adult disease, with significantly lower incidence in children. Myeloid sarcoma (MS), an extramedullary manifestation of AML, can occur in various tissues, though it is exceptionally rare in the orbit. The variable nature of MS complicates its diagnosis and treatment. In this report, we present the case of an eight-year-old boy who exhibited bilateral proptosis and an orbital mass. Initial investigations revealed thrombocytopenia and leukopenia. Imaging tests confirmed an expansive retroorbital lesion. Immunohistochemistry demonstrated positivity for CD34, CD117, and myeloperoxidase (MPO). The mass was ultimately diagnosed as MS. Subsequent bone marrow analysis confirmed AML. This case highlights that MS, although rare, can serve as an initial presentation of AML in pediatric patients. Epidemiology, diagnosis, treatment, and prognosis for MS were discussed.

## Introduction

Acute myeloid leukemia (AML) is a bone marrow neoplasm that is much more frequent in adults and rare in younger patients. Clinically, AML presents with signs and symptoms primarily resulting from the clonal expansion of myeloid precursors that infiltrate the bone marrow and disrupt hematopoiesis. Patients often present with fatigue and pallor, recurrent infections, and easy bruising and bleeding. The incidence of AML is 10 times lower for people less than 65 years compared to those older than 65 years, and it is exceptionally rare in pediatric patients [1]. The median age at diagnosis is 69 years (65-74) [2]. The rapid progression of AML and its complications make prompt recognition and intervention essential for its effective management.

Myeloid sarcoma (MS) is a rare manifestation that can develop in patients with AML. MS is a solid tumor composed of myeloblasts that infiltrates tissues outside of the bone marrow. MS is difficult to assess epidemiologically, but it has been described in 2.5-9.1% of all patients with AML [3,4]. It has been described in multiple locations, ranging from bone to gastrointestinal (GI) tract and soft tissue, but rarely in the periorbital region. Moreover, MS can be the first manifestation of AML, preceding it months or years before the actual disease. Given its variable and rare nature, MS can prove to be a challenge for diagnosis, particularly for healthcare providers who are less familiar with this neoplasm. In this article, we present a case of MS in the orbit as the first manifestation in an eight-year-old child who was later diagnosed with AML to showcase the importance of considering MS in the differential diagnosis of orbital masses in pediatric patients.

## Case presentation

Clinical findings

**Figure 1:**
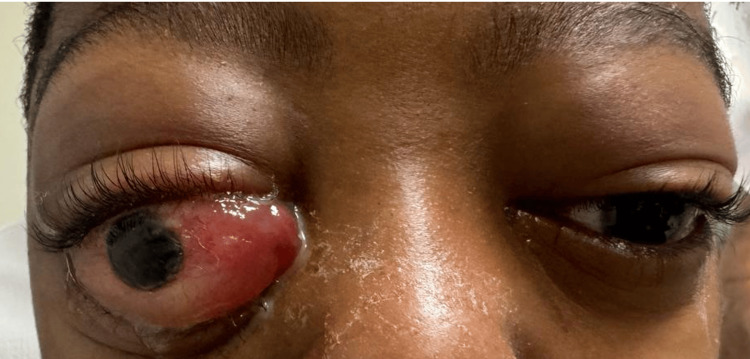
Right eye-predominant proptosis.

**Figure 2:**
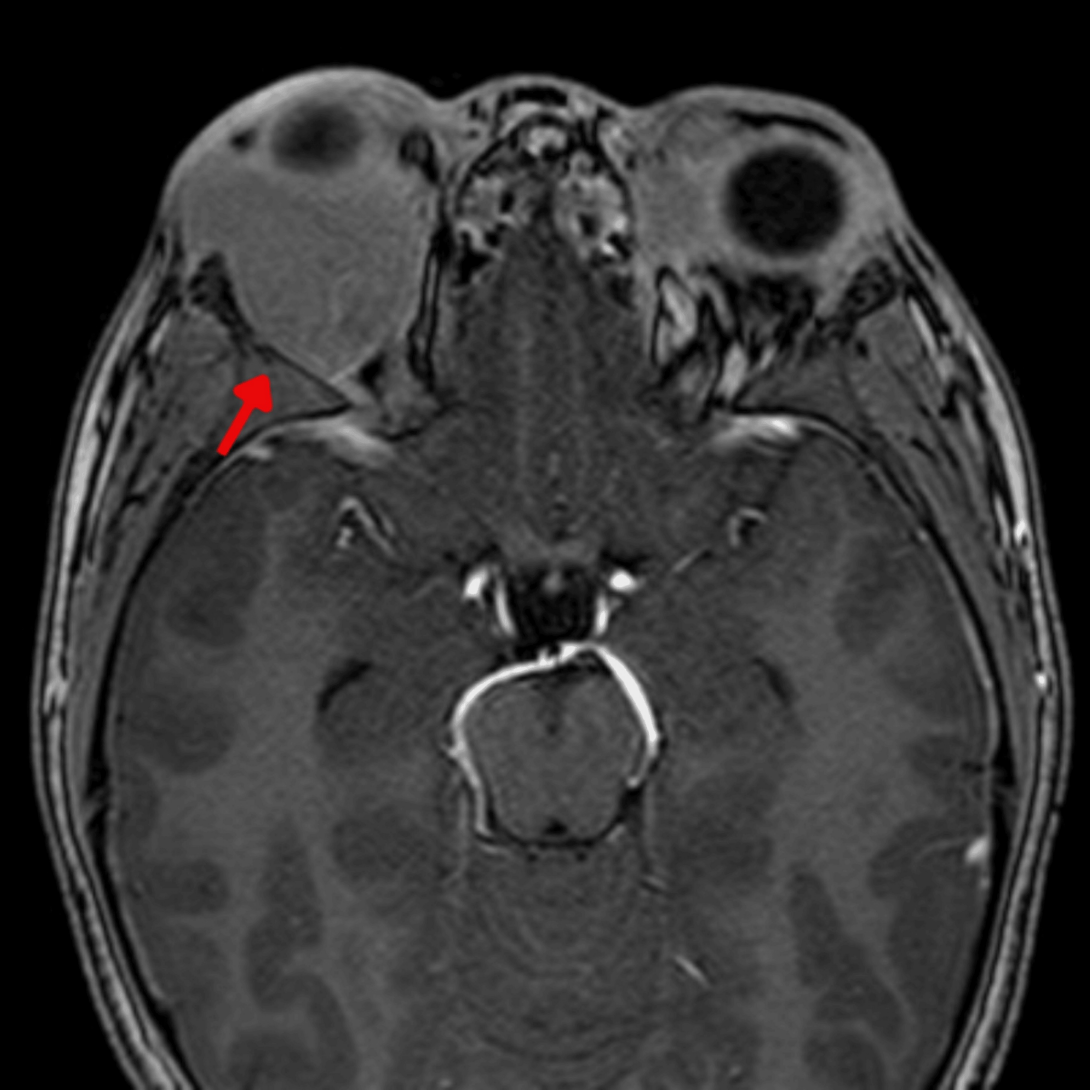
MRI in T1 phase with contrast showing an expansive right retroocular lesion (red arrow).

An eight-year-old boy with no significant past medical history was brought to the outpatient clinic with a one-month history of bilateral proptosis, predominantly affecting the right eye (Figure 1), as the only finding on the physical exam. An MRI confirmed the presence of an expansive iso-enhancing retroocular lesion that compressed the eye (Figure 2). A complete blood count showed thrombocytopenia and leukopenia, which, alongside the MRI findings, warranted a biopsy of the lesion.

Histopathology

**Figure 3:**
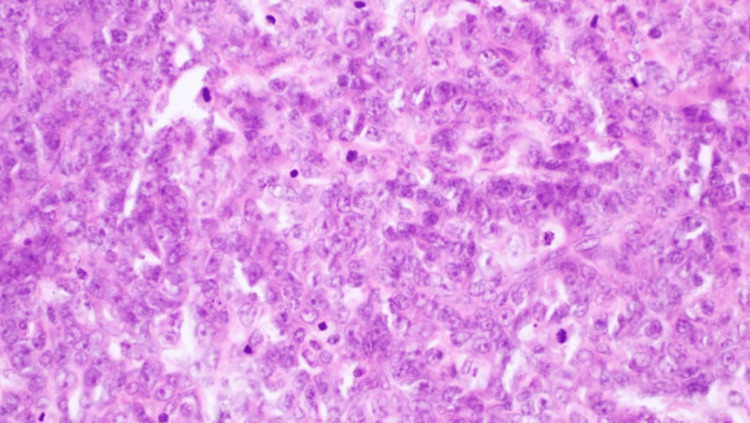
Numerous cells with a high nucleus-to-cytoplasmic ratio, prominent nucleolus, and mitotic figures.

**Figure 4:**
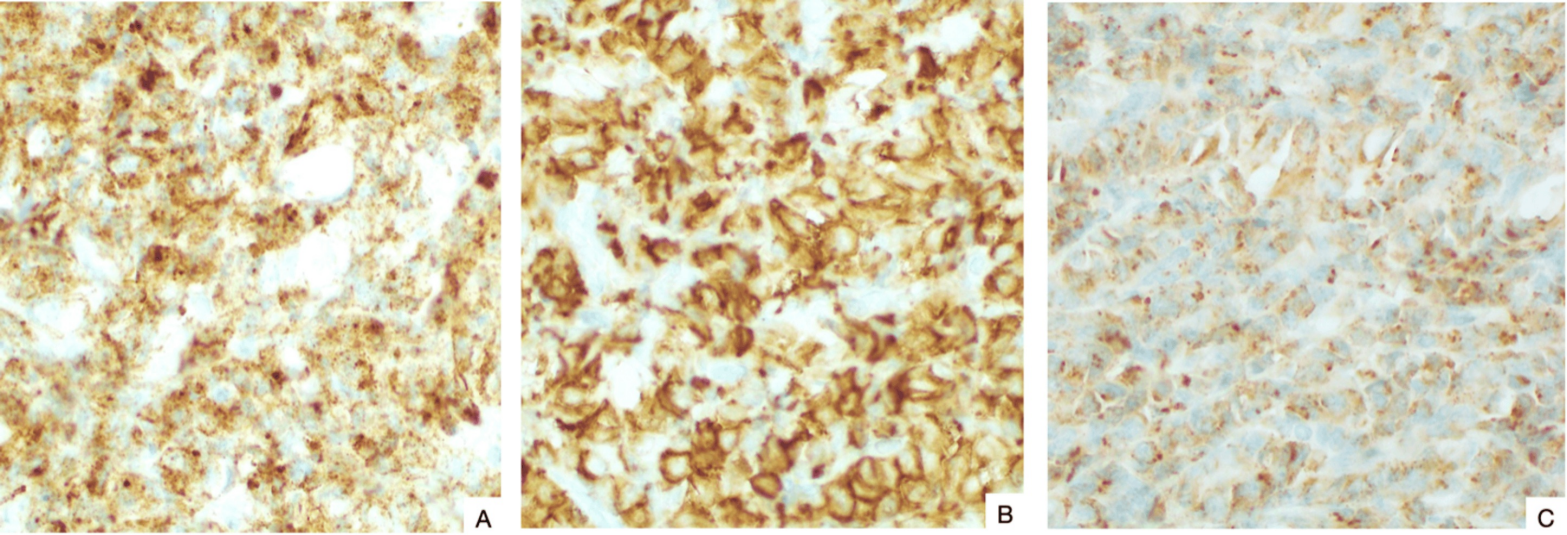
Immunohistochemical stains demonstrating positivity for (A) CD34, (B) CD117, and (C) MPO. MPO: myeloperoxidase.

Histological sections revealed a densely cellular tumor composed of sheets of atypical cells with a high nucleus-to-cytoplasmic ratio. Nuclei were irregularly round or ovoid with prominent nucleolus, open chromatin, and numerous mitotic figures. The cytoplasm was scant and eosinophilic. The cells were arranged diffusely, occasionally with perivascular distribution (Figure 3). Immunohistochemically, the tumor cells were positive for CD34, CD117, and myeloperoxidase (MPO) and focally positive for CD15 (Figure 4).

**Figure 5:**
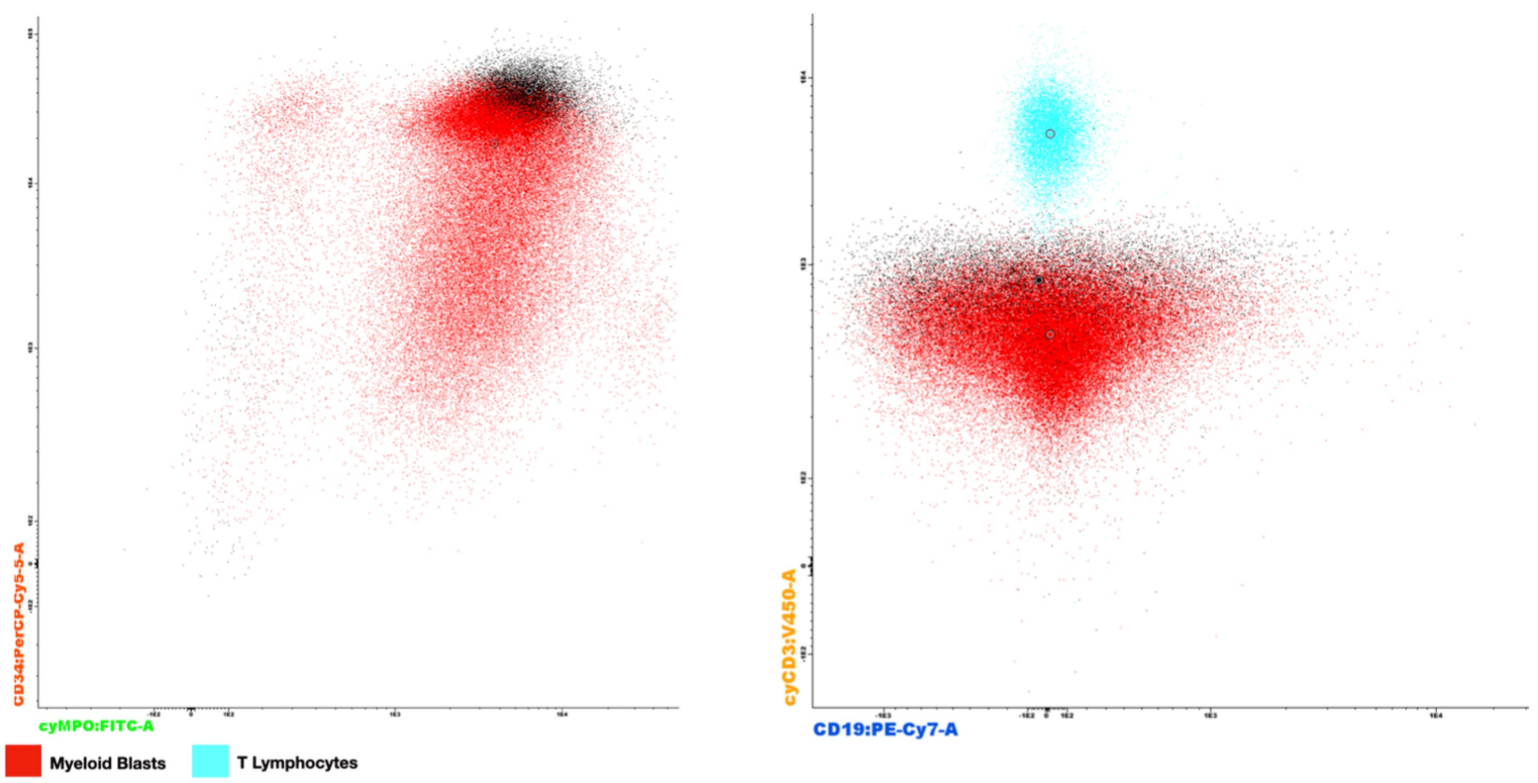
Flow cytometry showing a population of cells with high expression of MPO and CD34, while expressing relatively low CD3 and CD19, confirming their identity as myeloid blasts (red). MPO: myeloperoxidase.

Evaluation by the pediatric hemato-oncology team was done, and a decision was made to perform a bone marrow biopsy and aspirate. Flow cytometry revealed 57% myeloid blasts (CD45+(weak), CD34+, CD117-, CD64-, CD33+, CD13+) that lacked highly specific markers for lymphoid lineage (cyCD3-, CD19-, cyCD79a-, CD22-, CD7-, CD10-) but showed maturation into the neutrophilic lineage (cMPO++, CD45+, CD117-, CD33+, CD64-, CD36-, CD16-, IREM 2-) (Figure 5). Additionally, 8.4% of mature basophils were identified. The aspirate was ultimately reported as AML with maturation.

## Discussion

MS, also known as granulocytic sarcoma, or chloroma in earlier literature, is an extramedullary tumor composed of immature myeloid cells. It is a rare manifestation that can occur in various anatomic locations, including the orbit [3]. This lesion is often present in association with AML, as in this case, other myeloid disorders, or more rarely as an isolated entity. Every subtype of AML can present with MS. The pathogenesis of MS is unknown; some theories have been proposed, but none of them have been proven. Notably, when MS is diagnosed without any previous history, 80-90% of these patients are found to have underlying AML [5]. Moreover, even if AML is not diagnosed at that moment, the presentation of MS is a herald of this condition [6].

Epidemiologically, the age of onset varies based on the location of the tumor. The mean age of diagnosis for MS is 56.2 years, while the mean age of diagnosis for orbital MS is 8.8 years [7]. This younger age of onset may influence its clinical presentation and the treatment strategies taken. The anatomical distribution is variable, although the three most commonly documented sites of growth are connective/soft tissue (38.7%), skin or breast (15%), and the digestive system (11.7) [8,9]. Head and neck have a presentation rate of 7.5%, with the orbit being the most common site to be involved in this region, especially in patients younger than 18 years [10,11].

The presentation of MS is typically rapid, with its symptoms depending on the location of the lesion. Patients with orbital MS commonly complain of proptosis, pain, edema, and vision impairment [4]. As the differential diagnosis includes lymphoma, rhabdomyosarcoma, and metastatic lesions, distinguishing orbital MS is crucial for its prompt treatment. Although imaging tests (CT or MRI) are helpful in assessing differential diagnoses, biopsy and immunohistochemical analysis should always be used to confirm the diagnosis. MS typically exhibits a diffuse infiltrate of blast cells with a high nucleus-to-cytoplasmic ratio and expresses markers that are associated with AML, such as CD68, MPO, CD34, CD43, CD117, CD56, CD30, and CD99 [12,13].

The presentation and location of MS are important for the election of a treatment option. While there is no consensus on any type of regimen that should be used, it is widely recommended that polychemotherapy should be started regardless of bone marrow status. Even though there is no indication for radiation therapy, low doses can be used either for mass reduction of organ- or life-threatening MS or symptomatic relief [5,12]. As in AML, hematopoietic stem cell transplantation after remission seems to be the best option for improving outcomes and overall survival, although it is still associated with a high rate of recurrence (50%) [12,14].

The prognosis for MS seems to be variable, and most factors regarding it remain controversial. When MS presents concomitantly with AML, the prognosis appears to be better when compared to an isolated presentation [14,15], although other sources report no difference on it. It appears that anatomical location is related to overall survival (OS); it has been shown that locations such as reproductive organs and the digestive system have a median survival of 5.2 and 2.2 years, respectively, while nervous development of MS has an associated OS of ~8 months [8,9].

## Conclusions

AML is a rare condition in children. When it does occur, presenting as MS is notably uncommon, especially in an orbital location. This case illustrates the rare occurrence of AML presenting firsthand as MS in a pediatric patient, specifically within the orbital region. The findings from this case contribute to the existing literature by demonstrating that MS can precede AML by months or years, particularly in younger patients. Furthermore, it underscores that knowledge of the epidemiology and the possible manifestation of MS is paramount, as it can serve as an early indicator of underlying AML so proper diagnostic evaluations and management are employed promptly to improve patient outcomes.
